# Sex-Based Associations Between Education Level, EAT–Lancet Diet, and 20-Year Cardiovascular Risk: The ATTICA Study (2002–2022)

**DOI:** 10.3390/nu17172827

**Published:** 2025-08-30

**Authors:** Evangelia G. Sigala, Christos Pitsavos, Fotios Barkas, Evangelos Liberopoulos, Petros P. Sfikakis, Costas Tsioufis, Demosthenes Panagiotakos

**Affiliations:** 1 Department of Nutrition and Dietetics, School of Health Sciences and Education, Harokopio University, 17671 Athens, Greece; 2 First Cardiology Clinic, Hippokration Hospital, Medical School, National and Kapodistrian University of Athens, 15772 Athens, Greece; 3 Department of Internal Medicine, Medical School, University of Ioannina, 45500 Ioannina, Greece; 4 First Department of Propaedeutic Internal Medicine, Laiko General Hospital, Medical School, National and Kapodistrian University of Athens, 15772 Athens, Greece

**Keywords:** cardiovascular disease epidemiology, risk assessment, primary prevention, education, sustainable diets

## Abstract

**Background/Objectives**: To investigate the associations between educational attainment and 20-year cardiovascular disease (CVD) incidence, mortality, lifetime risk, and burden, and to explore the mediating role of healthy and sustainable dietary habits through a sex-specific lens. **Methods**: A total of 3042 CVD-free adults from the ATTICA Study were included at the 2001/2002 baseline. Educational level was treated as both continuous and ordinal variable. Adherence to the EAT–Lancet diet pattern (EAT-LDP) was assessed at baseline. Participants were followed for 20 years, with complete data on CVD outcomes available for 1988 individuals. Generalized structural equation and nested Cox regression models were used to estimate the direct and indirect effects between education attainment and 20-year CVD incidence. Moderation analysis was also conducted by incorporating interaction terms in Cox models. **Results**: An inverse educational gradient in CVD risk and burden was observed, particularly among females for lifetime risk estimates. Each additional year of education was associated with higher EAT-LDP adherence (β = 0.45, 95% CI: 0.40–0.50) and increased odds of physical activity (OR: 1.01, 95% CI: 1.00–1.01). These behaviors mediated part of the relationship between education and long-term CVD incidence. Among females, the cardioprotective role of EAT-LDP adherence was more evident at lower educational levels, suggesting potential effect modification. **Conclusions**: Educational disparities in long-term CVD outcomes are partly mediated by sustainable dietary habits. These findings highlight the need for gender-responsive and equity-focused strategies in cardiovascular prevention.

## 1. Introduction

According to the Global Burden of Disease Study, from 2010 to 2021, age-standardized disability-adjusted life years (DALYs) attributable to cardiovascular disease (CVD) declined approximately by 14.0%, with comparable reductions noted among males (−14%) and females (−15%) [1]. Discrepancies were observed upon consideration of the Socio-demographic Index (SDI), which integrates per capita incomes, fertility rates, and mean years of schooling. Specifically, the decline in CVD burden was markedly attenuated in low-SDI regions (females: −7%; males: −5%) relative to the more pronounced reductions observed in high SDI regions (both sexes: −17%). Alarmingly, between-SDI differences became more pronounced over the decade, particularly among females, with the low-to-high SDI DALYs ratio escalating from 2.8 in 2010 to 3.1 in 2021, while the respective ratio for males rose from 1.9 to 2.1. These trends underscore the vulnerability of females living in these socio-economically disadvantaged locations.

Mounting evidence has identified social determinants of health, such as education, income, and occupation, as fundamental contributors to these inequities that shape health outcomes [2]. Among these, educational attainment has emerged as one of the strongest predictors of health outcomes, including CVD. Epidemiological studies [3,4,5] and meta-analyses [6,7] have documented robust inverse associations between education and CVD outcomes and its and risk factor [7,8,9]. These associations are explained by lifestyle, psycho-social, and structural factors [4,8,10]. Specifically, educational attainment shapes dietary behaviors, influencing the likelihood of adherence to health-promoting and environmentally sustainable dietary patterns. In recent years, such patterns, like the Mediterranean dietary pattern (MDP) [11] and the EAT–Lancet Diet Pattern (EAT-LDP) recommended of the Lancet Commission [12], have gained substantial attention as dual-purpose strategies for promoting health and mitigating environmental degradation, while ensuring equity, inclusion, affordability, accessibility, and cultural acceptability [11,13].

Nevertheless, substantial gaps remain in the existing literature, particularly concerning studies with long-term observational period, adjustment for competing risks, and representation from Mediterranean populations. Moreover, sex-specific analyses remain limited, despite growing recognition of their importance in CVD. Hence, to address these gaps, this study aimed to investigate the complex interplay between educational attainment, adherence to healthy and sustainable dietary patterns, and long-term CVD outcomes, using sex-stratified data from the 20-year ATTICA cohort in Greece. The EAT–LDP was selected as an a priori, sustainability-aligned framework and was operationalized as a 0–42 score, enabling a prespecified evaluation of the education-diet interplay with 20-year CVD risk in this Mediterranean cohort. Moreover, this perspective recognizes that sex (defined by biological and physiological traits) and gender (shaped by social norms and culturally embedded roles) interact with socio-economic inequities in ways that collectively influence health-related behaviors, healthcare access, and long-term health trajectories [14].

## 2. Materials and Methods

### 2.1. Ethical Compliance and Protection of Personal Data

The ATTICA Study was designed and conducted in full accordance with the principles guiding biomedical research involving human subjects, as outlined in the Declaration of Helsinki and its amendments. Ethical approval was obtained from the Ethics Committees of the National and Kapodistrian University of Athens (#017/1 May 2001) and Harokopio University (#38/29 March 2022). Prior to enrollment, all individuals received detailed information about the study’s objectives and procedures and subsequently provided signed informed consent. Throughout the study, strict protocols were followed to ensure the confidentiality and the security of personal data.

### 2.2. Design, Setting, and Participant Recruitment

The ATTICA Study is a prospective, population-based cohort spanning a two-decade observational period, designed to investigate the multifactorial associations between socio-demographic characteristics, clinical and biochemical profiles, anthropometric parameters, and lifestyle behaviors in relation to CVD outcomes. Methodological aspects and protocols have been thoroughly documented in previous publications [15,16,17,18]. Data collection and clinical evaluations were conducted by a multidisciplinary team of trained healthcare professionals.

Baseline participant recruitment was carried out between 2001 and 2002 using multistage, stratified random sampling to ensure representativeness of the adult population (≥18 years) residing in the Attica region of Greece. Stratification was performed according to sex (female/male) and five age brackets, based on the demographic structure reported in the 2001 National Population-Housing Census. A total of 27 municipalities or communes were included, capturing both urban (78%) and suburban (22%) areas. Individuals with a history of CVD, cancer, or other inflammatory conditions, as well as those who had undergone surgery within the past week, were excluded. Out of the 4056 eligible subjects reached to participate, 3042 volunteers (*n* = 1528 females 18–89 years old; *n* = 1514 males 18–87 years old) were enrolled in the 2001/2002 baseline assessment, corresponding to a participation rate of 75% [17].

Successive follow-up evaluations were conducted at 5 [18], 10 [16], and 20 [15] years after the 2001/2002 baseline, yielding participation rates of 69%, 85%, and 71%, respectively. Surviving cohort members were re-approached to arrange face-to-face follow-up. For deceased volunteers, mortality data (date and cause of death) were obtained from relatives and cross-validated with official death registration certificates. At the 20-year follow-up, complete CVD endpoint data were available for 1988 individuals (50.4% females, 44 ± 14 years; 49.6% males, 46 ± 13 years) [15]. The age and sex distributions of this subsample did not differ significantly from those of the 2001/2002 baseline cohort (*p*-values > 0.80), suggesting minimal attrition bias.

### 2.3. Data Collection at 2001/2002 Baseline

Baseline evaluations included the assessment of socio-demographic factors, lifestyle characteristics, familial predisposition to CVD, medical and medication history, arterial blood pressure measurements, anthropometry, and fasting blood collection [17].

#### 2.3.1. Ascertainment of Socio-Demographic Factors, Including Educational Attainment

Socio-demographic data were collected using a structured, self-administered questionnaire. The collected information included age (calculated from date of birth), biological sex (as a binary variable: male/female), marital status, number of offspring, occupation, educational attainment, and financial status (mean annual income of the past three years). Socio-economic status was determined as a composite index combining educational level and financial status, as it has been previously described [15,16]. Specifically, educational attainment was evaluated both as a continuous variable, representing total years of formal schooling, and as a three-level ordinal variable. Specifically, educational level was classified as: (a) “low,” including individuals with ≤9 years of schooling, i.e., those who had completed comprehensive, trade, or technical school; (b) “medium,” defined as 9–14 years of schooling, comprising participants who had attended or completed upper secondary education or vocational/trade college; and (c) “high,” referring to participants with >14 years of schooling, i.e., those who had attended or graduated from tertiary education, such as a university or equivalent institution [16].

#### 2.3.2. Assessment of Other Explanatory Variables

Lifestyle exposures assessment included smoking status, dietary habits, and physical activity. Smoking status was dichotomized as “never” (no history of tobacco use) or “ever” smokers. The latter category involved former or current smokers. Usual dietary intake was assessed using the validated 156-item semi-quantitative EPIC-Greek food frequency questionnaire (FFQ) [19], supported by photographs to aid portion size estimation. Volunteers reported the frequency of food and beverage consumption over the prior month, in daily or weekly servings. Alcohol intake was expressed in 100 mL wineglass equivalents, each containing 12 g of ethanol. Adherence to health-promoting and environmentally sustainable diets was evaluated using two a priori dietary indices: the EAT-LDP (range: 0–42) [20] and the MedDietScore (range: 0–55) [21]. Both scores emphasize the consumption of plant-based foods and fish and discourage the consumption of red, white, and processed meats, full-fat dairy products, and added sugars, while the MedDietScore incorporates alcohol intake on a non-linear scale. Physical activity was evaluated utilizing the validated Greek version of the Short Form of the International Physical Activity Questionnaire (IPAQ-SF) [22]. Inactivity was defined as the absence of any reported recreational activity episode lasting at least 10 min.

Following standardized protocols, body weight, height, waist circumference (WC), and hip circumference were recorded using calibrated anthropometry equipment [17,23]. These measurements were subsequently used to derive body mass index (BMI), waist-to-height (WHtR), and waist-to-hip (WHR). Arterial blood pressure was assessed using a calibrated aneroid sphygmomanometer on the right arm positioned at a 45° angle, following a seated rest of 30 min. Three consecutive readings were recorded, and their average was used. Moreover, a single (World Health Organization) WHO-compliant reference laboratory conducted all laboratory assays [17,24], which included lipid and hematologic profiles, glucose metabolism parameters, hepatic and renal function, and biomarkers of inflammation and coagulation. Clinical status ascertainment was focused on diagnoses of hypertension (mean arterial blood pressure >140/90 mmHg or use of antihypertensive drugs), hypercholesterolemia (total serum cholesterol >200 mg/dL or use of lipid-lowering medication), and type 2 diabetes mellitus or diabetes mellitus (fasting plasma glucose ≥126 mg/dL or use of oral hypoglycemics or subcutaneous insulin treatment) [17]. For each condition, awareness categories were defined as follows: “aware” indicated a prior physician diagnosis and/or current disease-specific medication; “untreated but aware” indicated a prior diagnosis without current pharmacotherapy at baseline; and “unaware” indicated newly detected cases at baseline by study measurements without prior diagnosis or treatment.

### 2.4. Primary Endpoints Measures at the 2006, 2011/2012, and 2022 Follow-Up Examinations

The study outcomes included incident CVD events, encompassing fatal and non-fatal cases, as classified using the 9th and 10th revisions of the International Classification of Diseases (ICD-9 and ICD-10) by the WHO [15,16,18]. Additionally, non-CVD-related mortality was systematically recorded to account for competing risks in the estimation of lifetime CVD risk.

### 2.5. Statistical Analysis

**Figure 1:**
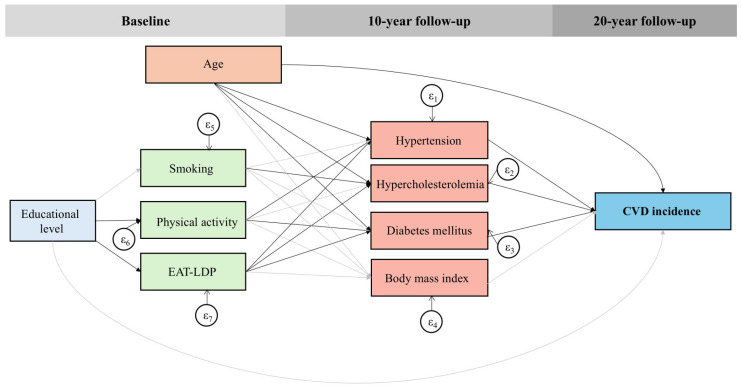
Generalized structural equation model (GSEM) exploring the complex pathways between education and long-term (20-year) CVD incidence.

Categorical variables are summarized as relative frequencies (%), and continuous variables are presented as means with standard deviations (SD) or medians with interquartile ranges, depending on their distributions. Crude incidence and mortality rates were calculated as the number of incident CVD events divided by the number of participants at risk at each follow-up. Participants were followed from baseline until the first CVD event, last confirmed contact, non-CVD death, or study end. Those without CVD were right-censored; non-CVD deaths were treated as competing events in the lifetime risk analyses. Specifically, remaining or residual lifetime CVD risk was estimated from baseline up to 80 years of age, due to limited data availability beyond this age. Stratified estimates by sex and educational level were computed for the age indices of 40, 50, and 60 using a modified Kaplan–Meier life table method adjusted for competing risks attributed to non-CVD mortality [25,26]. The burden of CVD was expressed in DALYs, representing the sum of lost life years owing to premature CVD mortality and the years lived with CVD-related disability, where lost life years due to premature CVD mortality were calculated as the number of CVD deaths multiplied by the remaining standard life expectancy at the age of death and the years lived with CVD-related disability for each condition were computed as the number of years lived with that condition multiplied by the corresponding disability weight. Between-group comparisons were performed using the chi-square test for categorical variables, ANOVA for normally distributed continuous variables, and the Kruskal–Wallis test for skewed numerical variables. The log-rank test was used to compare cumulative incidence rates. To assess whether the associations between lifestyle factors and long-term (20-year) CVD incidence differed by educational level, a moderation analysis was conducted using fully adjusted Cox proportional hazards models with interaction terms. Specifically, three interaction terms between educational attainment and smoking status, EAT-LDP, and physical activity were introduced in the model, while controlling for age, hypertension, hypercholesterolemia, diabetes mellitus, and WHtR, as well as the main effects of education and each lifestyle factor. To explore mediation pathways, the association between educational attainment and 20-year CVD incidence was further evaluated using nested Cox regression models, after verifying proportional hazards assumption. Hazard ratios (HRs) and 95% confidence intervals (CIs) were estimated following hierarchical adjustment for blocks of CVD predisposing factors, introduced sequentially based on their presumed effect size, to quantify the direct and indirect effects of education through lifestyle mediators. Generalized structural equation modeling (GSEM) was also employed to explicitly model the hypothesized pathways linking education, mediators, and 20-year incident CVD (Figure 1). The binary outcome (i.e., 20-year CVD incidence) was modeled using a binomial distribution with a logit link function. All tests were two-sided, and statistical significance level was set at *p*-value < 0.05. Analyses were conducted using STATA version 18.0 (StataCorp, College Station, TX, USA).

## 3. Results

### 3.1. Crude Analysis of CVD Outcomes by Educational Level

Between the 2001/2002 baseline and the 2022 follow-up, 32% of participating females and 40% of males experienced a fatal or non-fatal CVD event (*p* for sex difference: <0.001). Substantial discrepancies were also observed across educational levels, both in the overall sample, as well as within and between sexes (Table 1).

In the total cohort, individuals with lower educational attainment exhibited a markedly higher cumulative incidence of CVD (62.9%) over the 20-year period, compared to their medium (30.9%) or high (26.6%) educated counterparts (*p* for trend: <0.001). This educational gradient remained evident when analyses were stratified by sex (*p*-values for trend: <0.001). A similar pattern was observed for CVD mortality. Specifically, low-educated females experienced a fivefold greater CVD mortality rate than those with >9 years of schooling (*p* for trend: <0.001), while among males the corresponding difference was even more pronounced (*p* for trend: <0.001). Intriguingly, although a higher educational level seems to exert cardioprophylactic effects, the onset of first CVD events was observed significantly earlier among individuals with medium and high educational levels compared to those with lower education (*p*-values for trend: <0.001). Further sex-stratified analyses revealed that among subjects with medium (*p* for sex difference: 0.003) and high (*p* for sex difference: 0.001) educational levels, males exhibited a significantly greater 20-year CVD incidence compared to females. Additionally, across all educational levels, females demonstrated lower mortality rates than males over the 20-year observation period (*p*-values for sex differences: low and medium levels: <0.001; high level: 0.004). No other sex-specific discrepancies were found.

Estimates of remaining or residual lifetime CVD risk also demonstrated a downward trend with increasing educational level, most notably among individuals of both sexes at the age index of 40 years (*p*-values for trend: <0.05) and among females at the age index of 50 (*p* for trend: 0.002). Within each educational level, sex-specific analyses showed that males had significantly higher lifetime risk for developing CVD up to the age of 80 years when measured from age indices of 40 (*p*-values for sex differences: <0.001) and 50 years (*p*-values for sex differences: low level: 0.026; medium level: 0.013; high level: <0.001). However, this sex difference attenuated at the index age of 60, indicating comparable lifetime CVD risk between males and females who had remained CVD-free up to that point. Regarding CVD burden, the number of DALYs attributed to CVD was significantly lower among low-educated participants, particularly among females (*p* for trend: <0.001).

### 3.2. Differences on Baseline CVD Predisposing Factors by Educational Level

Table 2 describes individuals’ baseline socio-demographic characteristics, major clinical risk factors, biomarkers, anthropometric indices, and lifestyle habits across three educational levels, in the total sample and by sex. Participants with lower educational attainment were significantly older, economically disadvantaged, and more frequently classified within the low socio-economic class (*p*-values for trend: <0.001). They also exhibited higher prevalences of hypercholesterolemia and diabetes mellitus, elevated fibrinogen levels, less favorable WHtR and WHR, greater energy intake relative to their basal metabolic rates, and were less likely to adhere to healthy and sustainable dietary patterns, as assessed via the EAT-LDP and the MedDietScore (*p*-values for trend: <0.05). On the other hand, untreated but aware cases of hypertension, hypercholesterolemia, and diabetes mellitus were more frequent among higher-educated participants. Educational gradients were evident in both sexes, but they were more pronounced among females, especially regarding hypertension, overweight/obesity, central adiposity, WC, and high-sensitivity C-reactive protein (hs-CRP) levels (*p*-values for trend: <0.001). Smoking followed an inverse J-shaped pattern, with the highest prevalence in the medium education group, especially among females (*p*-values for trend: <0.05).

Sex-specific differences were observed across each educational level. Among participants with low education, females were older than males (*p* for sex difference: 0.037), while in the medium (*p* for sex difference: 0.016) and high (*p* for sex difference: <0.001) education levels, males were older. Across all education levels, females were more likely to self-report low income than males (*p*-values for sex differences: <0.05). Regarding clinical status, males with medium and high educational levels had higher hypertension and hypercholesterolemia prevalences than females (*p*-values for sex differences: <0.001); while among those with ≤9 years of schooling, diabetes mellitus was more common in males (*p* for sex difference: 0.028). Females with low and medium education had significantly higher fibrinogen levels than males (*p*-values for sex differences: <0.001), and among highly educated individuals, females had greater hs-CRP levels (*p* for sex difference: 0.003). Statistically significant between-sex differences were also observed in overweight/obesity and central adiposity across all educational levels (*p*-values for sex differences: <0.05). Males with >9 years of schooling had higher BMI than females (*p*-values for sex differences: <0.001), whereas males across all educational levels exhibited significantly greater WC, WHtR, and WHR values than their female counterparts (*p*-values for sex differences: <0.001). Among high-educated participants, females had higher energy intake adjusted for BMR than males (*p* for sex difference: 0.004). In terms of adherence to a healthy and sustainable dietary pattern, low-educated females had lower adherence to the EAT-LDP compared to males (*p* for sex difference: 0.032), while highly educated females showed greater adherence to both the EAT-LDP (*p*-values for sex differences: continuous <0.001; categorical = 0.030) and the MDP (*p*-values for sex differences: <0.001 for both continuous and categorical variables) than males. Males were more likely to be current or former smokers than females (*p* for sex difference: <0.001), while physical inactivity was more common among low-educated females than males (*p* for sex difference: 0.010).

### 3.3. Moderation and Mediation Analysis of the Role of Education on the 20-Year CVD Risk

The potential effect modification of educational attainment on the association between lifestyle factors and 20-year CVD incidence was investigated by incorporating interaction terms into fully adjusted Cox proportional hazard models applied to the total sample and sex-stratified subgroups. In the total sample and among males, none of the interaction terms reached statistical significance (*p*-values for interactions: >0.05). However, among females, statistically significant interaction effects were observed between EAT-LDP and education, both when education was modeled as a continuous variable (HR: 1.02, 95% CI: 1.01–1.04; *p* for interaction = 0.029) and when analyzed categorically, specifically for the high education level (HR: 1.31, 95% CI: 1.01–1.69; *p* for interaction = 0.038), indicating a potential modifying effect of higher education on the cardioprotective association of EAT-LDP among females. Notably, the main effects of EAT-LDP were not statistically significant in the latter model, suggesting that, for females, the association between EAT-LDP and 20-year CVD risk is conditional on educational level. Specifically, these findings imply that EAT-LDP may confer differential effects on 20-year CVD risk across educational levels, with a potential cardioprotective role among lower-educated females but a diminished or ceiling effect among those with higher educational attainment.

To further disentangle the intricate pathways linking educational attainment with long-term CVD incidence, nested Cox proportional hazards models were constructed (Table 3). In the unadjusted model (Model 1), each additional year of formal education was associated with a 19% (HR: 0.81, 95% CI: 0.77–0.84) and an 11% (HR: 0.89, 95% CI: 0.86–0.93) reduction in 20-year CVD risk among females and males, respectively. These associations were attenuated upon further adjustments for age, clinical risk factors, WHtR, and lifestyle-related behaviors, suggesting that these factors may partially mediate the relationship between education and long-term CVD risk. Moreover, in the fully adjusted model (Model 4), among females, adherence to the EAT-LDP emerged as an independent cardioprotective factor. Specifically, each 1-unit increase in the EAT-LDP was associated with a 15% (HR: 0.85, 95% CI: 0.79–0.92) lower risk of experiencing a fatal or non-fatal CVD event over a 20-year period. A comparable inverse association (HR: 0.84, 95% CI: 0.78–0.90) was observed among males.

### 3.4. Generalized Structural Equation Model (GSEM)

As illustrated in Figure 1 and Table 4, educational attainment was not directly associated with 20-year CVD incidence (odds ratios (OR): 0.98, 95% CI: 0.92–1.05); however, it exerted indirect effects through lifestyle-related and clinical mediators. Specifically, each additional year of formal education was significantly associated with higher adherence to the EAT-LDP (β = 0.45, 95% CI: 0.40–0.50) and increased odds of physical activity engagement (OR: 1.01, 95% CI: 1.00–1.01). In turn, EAT-LDP adherence was inversely associated with clinical CVD risk factors (*p*-values < 0.05), all of which were strong predictors of 20-year CVD incidence (*p*-values < 0.001).

## 4. Discussion

Although previous studies have assessed the association between educational attainment and CVD incidence and mortality, education has predominantly been treated as a proxy for socio-economic status [10]. Longitudinal studies with long-term follow-ups, competing risk-adjusted lifetime risk estimates, or sex-specific analyses in Mediterranean populations remain scarce in the literature. To address these gaps, the associations between educational attainment and CVD outcomes were examined in the ATTICA Study, a representative cohort from Greece monitored over a 20-year observational period (2002–2022), yielding intriguing insights.

### 4.1. Main Epidemiological Findings

The present findings extend a previous report from the ATTICA study, that has highlighted the role of education level in the incidence of CVD, without studying, however, the critical role of sex and dietary habits [16]. In detail, a pronounced educational gradient in 20-year CVD incidence and mortality was observed, with individuals of lower educational level experiencing significantly higher fatal or non-fatal event rates. Specifically, participants with low educational level had nearly 2.5- and 5-times higher incidence and death rates, respectively, than their high-educated counterparts. Nevertheless, the onset of CVD occurred earlier in subjects with >9 years of schooling, potentially reflecting differential cohort exposures or healthcare-seeking behaviors. For example, in this study, individuals with higher educational attainment were more likely to be aware of diagnoses of major clinical risk factors yet remained untreated. Additionally, this finding may partly be explained by the sex-centric patterns observed among individuals with >9 years of schooling, wherein the male-to-female 20-year incidence rate ratios were >1. Males have been shown to manifest earlier CVD events in a previous publication of the ATTICA Study [31], whereas other studies have revealed that females are more likely to adopt primary prevention measures. Lifetime risk analyses further underscored the vulnerability of less educated individuals, and this gradient was mainly observed in females. However, males consistently demonstrated greater lifetime CVD risk within all educational strata compared to females, with sex differences attenuating beyond the age of 60. Paradoxically, lower DALYs estimates were estimated among low-educated subjects, possibly reflecting premature mortality.

### 4.2. The Pathways Between Educational Attainment and CVD Outcomes

In accordance with the findings of the present analysis, similar relationships have been reported in the existing literature. In a meta-analysis of 72 prospective and retrospective studies conducted across Europe, the USA, and Asia, subjects with low and medium educational levels experienced significantly elevated risks for various CVD endpoints compared to their highly educated counterparts [6]. Specifically, pooled risk ratios (RR) for the low versus high educational level were estimated at 1.50 (95% CI: 1.17–1.92) CVD, 1.39 (95% CI: 1.26–1.54) for CVD deaths, 1.36 (95% CI: 1.11–1.66) for coronary heart disease (CHD), and 1.23 (95% CI: 1.06–1.43) for stroke. A comparable, albeit attenuated, pattern was also observed for medium education levels, suggesting a dose–response effect. Similar gradients have been observed in low- and middle-income countries. For example, the WHO STEPS national surveys indicated that individuals who had attended or completed only primary education exhibited a higher 10-year CVD risk than their counterparts with tertiary educational level (Iraq: β = 2.61, 95% CI: 0.90–4.32; Brunei Darussalam: β = 2.62, 95% CI: 1.91–3.32) [32]. Additionally, the Korean National Health and Nutrition Examination Survey (KNHANES) cohort, which included 48,190 participants, reported increased CVD incidence among individuals with only primary education (HR: 1.71, 95% CI: 1.31–2.24) compared to university graduates [4]. Likewise, other authors revealed higher lifetime CVD risk among those with low educational attainment [3,8] and a shorter lifespan prior to incident event [8]. Beyond confirming a strong inverse relationship between education and CVD, the meta-analysis of 116 cohort studies by Backholer et al. [7] identified a more pronounced effect of education in females (RR: 1.66, 95% CI: 1.43–192) than in males (RR: 1.42, 95% CI: 1.25–1.63) [7], yielding a female-to-male ratio of relative risks (RRR) of 1.18 (95% CI: 1.03–1.36). In Italy, 132,686 adults were enrolled to the two waves of National Health Interview Surveys and followed up for ten years for incident CVD [33]. The analysis revealed that individuals with only primary school education had a 21% (95% CI: 1.12–1.33) greater risk of CVD among males and a 41% (95% CI: 1.27–1.63) higher risk among females compared to those with tertiary education, underscoring the persistence of the sex-specific educational gradient in CVD risk, within a Mediterranean high-income setting. Supportive evidence from US cohort studies further reinforces these findings [8]. Specifically, sex-stratified analyses incorporating competing risk models revealed a clear inverse educational gradient in CVD risk among both females and males, with a more pronounced effect observed in females at the lowest educational level. Notably, males with less than a high school education exhibited a 58% (95% CI: 1.38–1.80) higher risk of CVD events, while females in the same educational category had a 70% (95% CI: 1.49–1.95) greater risk, compared to college graduates. Nonetheless, among females who had completed tertiary education, no significant elevation in CVD risk was observed (HR: 0.98; 95% CI: 0.83–1.15), suggesting a potential ceiling effect in the cardioprotective benefits of higher education. Additionally, consistent with the present study, lifetime risks of CVD were found to be higher for males than females across all educational levels [3].

Educational attainment CVD risk through intertwined lifestyle-related, psycho-social, and structural mechanisms [8,10]. At the individual level, lower educational attainment is consistently associated with a higher prevalence of adverse lifestyle habits, including smoking, sedentarism, and suboptimal dietary patterns, which, in turn, foster hypertension, hypercholesterolemia, diabetes mellitus, and obesity [4,5,7,8,9]. In the present study, educational attainment was positively associated with adherence to healthy and sustainable diets, as well as a greater likelihood of engaging in physical activity, both of which were linked to reduced 20-year CVD risk via conventional clinical risk factors’ pathways. However, studies have unveiled that these behavioral mediators explain only part of the observed association [8,34]. Education is directly linked to vocational opportunities, income, enhanced health literacy, and thus, increased access to healthcare services [5,8,10]. Specifically, in this study, individuals with low educational attainment were more likely to be older, have lower income, belong to a disadvantaged socio-economic stratum, and display a more adverse cardiometabolic risk profile. Educational gradients in risk were evident in both sexes. Psycho-social stressors, such as job strains, depressive symptoms, and chronic stress, which are more prevalent in the less educated adults [4,5,8,10], contribute to elevated allostatic load and systemic inflammation [9]. Moreover, structural barriers and social discrimination, manifested through barriers in healthcare access and sustained socio-economic adversities, further amplify health inequities and CVD risk, particularly among the least educated [4]. These inequities are especially pronounced among females with low educational attainment, who face disproportionately elevated CVD risk compared to males with similar schooling, likely due to delayed diagnosis, gender-based barriers in healthcare, and under-treatment [7]. Moreover, sex-specific pathways, including heightened stress reactivity, reduced social capital, and caregiving responsibilities, may intensify CVD vulnerability in low-educated females.

### 4.3. The Interplay of Education, Healthy and Sustainable Dietary Habits, and CVD

In this study, the mediation analysis provides evidence that adherence to a health-promoting, sustainable dietary pattern mediates the relationship between educational attainment and long-term CVD incidence. Specifically, each additional year of education was significantly associated with higher adherence to a sustainable diet by a factor of 0.45 and increased odds of engaging in physical activity by 1.01. Moreover, educational attainment emerged as a potent modifier of the diet-CVD relationship among females. This finding either highlights a diminishing or ceiling effect on the relationship of healthy and sustainable dietary habits to CVD risk among high-educated females or warrants further exploration because of potential residual confounding.

Further findings from a national health survey conducted in Greece are in alignment with these results, with individuals with primary education having 3.80 (95% CI: 3.25–4.46) times greater odds of low MDP adherence compared to subjects with a tertiary educational level [35]. In another cross-sectional study among Portuguese adults, higher educational attainment (secondary education: β = 4.24, 95% CI: 0.65–7.84; tertiary education: β = 5.28, 95% CI: 2.37–9.28) was positively associated with adherence to a sustainable dietary pattern [36]. Similarly, it has been found that Portuguese individuals with secondary education had 43% (95% CI: 1.16–1.75) higher odds of low EAT-LDP adherence relative to college graduates [37]. Furthermore, this study highlighted sex-based differences, with men exhibiting higher odds of poor adherence than females. Nevertheless, in the MEDIET4ALL survey of over 4000 individuals, no significant discrepancies were observed between sexes; yet females reported higher intake of healthful, plant-based foods (*p* < 0.001) [38].

The CVD benefits of sustainable diets have been well-established. In a recent meta-analysis, greater adherence to the EAT-LDP was associated with reduced odds of major CVD (HR: 0.84, 95% CI: 0.80–0.89), CVD-specific mortality (HR: 0.83, 95% CI: 0.78–0.88), and a combined CVD risk reduction (HR: 0.84, 95% CI: 0.81–0.87) [39]. Likewise, in a sample of >200,000 female and male US adults, high adherence conferred a 17% (HR: 0.83; 95% CI: 0.78, 0.89) lower risk for CVD, 19% (HR: 0.81; 95% CI: 0.74, 0.88) for CHD, and 14% (HR: 0.86; 95% CI: 0.78, 0.95) for stroke [40]. Comparable findings from the Malmö Diet and Cancer Study showed risk reductions in CVD mortality (HR: 0.68, 95% CI: 0.54–0.84) [20], CHD (HR: 0.80, 95% CI: 0.67–0.96) [41], heart failure (HR: 0.93, 95% CI: 0.88–0.97) [42], and atrial fibrillation (HR: 0.84, 95% CI: 0.73–0.98) [43] 20–30 years post-baseline. Findings from 55,016 middle-aged adults living in Denmark showed an even stronger inverse relationship with subarachnoid hemorrhage risk (HR: 0.30, 95% CI: 0.12–0.73) [44]. In another sample of apparently healthy Black females, the highest adherence to this pattern was also linked to reduction in CVD deaths 18 years later [45]. Nonetheless, not all studies have observed significant associations [46,47]. Apart from the cardioprophylactic outcomes, adherence to EAT-LDP also aligns with environmental sustainability targets, being associated with lower greenhouse gas emissions and land use, albeit with a higher blue water footprint [48].

### 4.4. Strengths and Limitations

The ATTICA Study’s longitudinal design, incorporating three follow-up assessments over a 20-year observational time window, offers a robust framework for evaluating the long-term trajectory of CVD and its determinants. However, several limitations should be acknowledged. Educational attainment was assessed only at the 2001/2002 baseline and not updated prospectively throughout the study, possibly resulting in misclassification among younger participants who may have advanced their education thereafter. Nevertheless, the proportion of participants below 26 years old was relatively small to have a significant effect on the estimated associations. Additionally, reliance on self-reported data for lifestyle factors may be prone to measurement errors, partly attributed to recall bias. Nonetheless, the questionnaires employed had been previously validated within a Greek population and administered by trained health professionals to enhance data reliability. Financial status plays an intriguing role in the associations between social class and CVD outcomes; in this study, financial status was incorporated through socio-economic status, as previous analyses of the ATTICA study have shown that it has a strong collinearity with education level [16,18]. While extensive covariate adjustment was performed, residual confounding cannot be entirely excluded. In particular, the lack of dynamic, life-course data on critical social determinants, such as food security, healthcare access, and housing quality, limits the capacity to fully elucidate the socio-economic pathways influencing long-term CVD outcomes.

## 5. Conclusions

The present findings highlight an inverse gradient between educational attainment and long-term CVD risk. Education remains a robust social determinant of health that can guide preventive future strategies, especially among disadvantaged groups. Sex-specific disparities further highlight the need for sex-responsive, equity-oriented public health interventions, including the promotion of healthy and sustainable dietary habits.

## Figures and Tables

**Table 1:** Total and sex-specific distributions of unadjusted 20-year CVD outcomes, lifetime risk estimates, and disease burden (in DALYs) across educational levels among ATTICA Study participants (*n* = 1988).

	Total Sample				Females				Males			
Epidemiological Indices	Low (*n* = 422)	Medium (*n* = 847)	High (*n* = 719)	*p*-Value for Trend	Low (*n* = 225)	Medium (*n* = 431)	High (*n* = 345)	*p*-Value for Trend	Low (*n* = 197)	Medium (*n* = 416)	High (*n* = 374)	*p*-Value for Trend
Age at first CVD event, years	75 (15)	65 (16)	61 (14)	<0.001	75 (12)	65 (12)	59 (24)	<0.001	72 (19)	65 (18)	61 (13)	<0.001
20-year CVD incidence, %	62.9	30.9	26.6	<0.001	62.7	26.5	19.1	<0.001	62.9	35.6	33.4	<0.001
≤35 years old	10.7	5.6	4.1		0	3.4	3.7		14.3	8.3	4.7	
35–45 years old	6.7	6.2	7.6		5.4	3.1	4.5		8.1	10.0	10.1	
45–55 years old	51.6	53.6	52.1		40.3	55.0	41.2		64.4	52.4	59.2	
55–65 years old	97.8	95.0	96.3		96.5	90.2	91.3		100.0	100.0	100.0	
>65 years old	100.0	100.0	100.0		100.0	100.0	100.0		100.0	100.0	100.0	
20-year CVD mortality, %	11.2	3.2	2.2	<0.001	5.4	0.9	0.8	<0.001	18.2	5.7	3.6	<0.001
Lifetime CVD risk, % (95% CI)												
40–50 years old	72.3 (70.4, 74.2)	71.0 (69.7, 72.2)	69.3 (67.9, 70.7)	0.050	66.6 (64.0, 69.3)	65.7 (64.0, 67.4)	61.8 (59.6, 63.9)	0.004	79.2 (77.4, 81.1)	76.9 (75.5, 78.4)	74.3 (72.8, 75.8)	0.001
50–60 years old	65.7 (63.9, 66.7)	65.8 (63.9, 66.7)	63.5 (61.9, 65.2)	0.091	63.7 (61.3, 66.2)	63.8 (61.7, 65.9)	58.6 (56.1, 61.1)	0.002	67.7 (65.2, 70.3)	66.8 (65.1, 68.6)	67.0 (65.1, 68.9)	0.684
60–70 years old	66.5 (64.5, 68.5)	65.3 (63.2, 67.4)	62.5 (58.8, 66.1)	0.186	66.2 (63.5, 68.9)	66.6 (63.7, 69.6)	64.1 (63.9, 70.0)	0.808	67.0 (63.9, 70.0)	64.0 (61.0, 66.9)	61.0 (56.2, 65.9)	0.070
CVD burden, DALYs (95% CI)	10.2 (9.1, 11.4)	13.4 (12.0, 14.8)	13.3 (11.7, 14.9)	0.006	9.7 (8.3, 11.1)	13.8 (12.0, 15.5)	14.5 (11.4, 17.5)	<0.001	11.0 (9.0, 13.0)	13.1 (11.0, 15.2)	12.7 (10.8, 14.6)	0.604

Non-normally distributed continuous variables are expressed as medians (interquartile ranges) and categorical variables are presented as relative frequencies (%). *p*-values were calculated using the log-rank test for 20-year incidence and mortality, and the Kruskal–Wallis test for continuous outcomes. Abbreviations: DALYs: disability-adjusted life years, 95% CI: 95% confidence interval.

**Table 2:** Total and sex-specific baseline socio-demographic, clinical, laboratory, anthropometric, and lifestyle characteristics across educational levels among ATTICA Study participants (*n* = 1988).

	Total Sample				Females				Males			
Variables	Low (*n* = 422)	Medium (*n* = 847)	High (*n* = 719)	*p*-Value for Trend	Low (*n* = 225)	Medium (*n* = 431)	High (*n* = 345)	*p*-Value for Trend	Low (*n* = 197)	Medium (*n* = 416)	High (*n* = 374)	*p*-Value for Trend
Age, years	54 (19)	43 (18)	41 (17)	<0.001	56 (19)	42 (18)	39 (17)	<0.001	52 (20)	44 (18)	43 (15)	<0.001
Financial status, % low income	77.7	62.1	33.8	<0.001	88.8	68.7	48.3	<0.001	66.4	55.6	21.6	<0.001
Socio-economic status, %				<0.001				<0.001				<0.001
Low class	81.0	0	0		83.2	0	0		78.9	0	0	
Middle class	19.0	94.6	13.1		16.8	95.5	18.5		21.6	93.7	8.6	
High class	0	5.4	86.9		0	4.5	81.5		0	6.3	91.4	
Residential setting, % urban	79.2	77.1	77.2	0.680	79.6	77.3	78.3	0.794	78.7	76.9	76.2	0.799
Hypertension, %	40.2	30.6	26.6	<0.001	41.0	22.6	13.9	<0.001	39.4	39.3	38.4	0.957
Treated	48.4	27.3	21.7		53.5	33.3	24.4		42.7	23.5	20.7	
Untreated but aware	51.6	72.7	78.3		46.5	66.7	75.6		57.3	76.5	79.3	
Unaware	0	0	0		0	0	0		0	0	0	
Hypercholesterolemia, %	56.6	39.9	37.3	<0.001	55.6	34.3	31.3	<0.001	59.0	45.7	42.9	<0.001
Treated	16.1	9.5	10.1		16.0	9.1	8.3		16.1	12.1	11.2	
Untreated but aware	83.9	90.5	89.9		84.0	90.9	81.7		83.9	87.9	88.8	
Unaware	0	0	0		0	0	0		0	0	0	
Diabetes mellitus, %	15.6	5.8	3.8	<0.001	12.0	4.9	2.3	<0.001	19.8	6.7	5.1	<0.001
Treated	51.5	40.8	22.2		59.3	47.6	25.0		46.2	35.7	21.1	
Untreated but aware	43.9	51.0	74.1		33.3	47.6	75.0		51.3	53.6	73.7	
Unaware	5.6	8.2	3.7		7.4	4.8	0		2.5	10.7	5.2	
Fibrinogen, mg/dL	328 ± 72	305 ± 65	306 ± 71	<0.001	340 ± 71	316 ± 66	307 ± 74	<0.001	312 ± 70	293 ± 63	305 ± 68	0.006
hs-CRP, mg/L	1.34 (2.11)	1.03 (1.85)	0.88 (1.80)	<0.001	1.62 (2.39)	0.88 (2.06)	0.75 (1.73)	<0.001	1.22 (1.77)	1.16 (1.66)	1.04 (179)	0.472
Overweight and/or obesity, %	71.7	54.9	52.0	<0.001	65.8	40.8	30.9	<0.001	78.5	69.5	71.6	0.069
Increased WC, %	64.0	51.5	48.6	<0.001	68.9	46.9	39.1	<0.001	58.4	56.3	57.4	0.876
BMI, kg/m^2^	27.6 ± 4.6	26.1 ± 4.5	25.8 ± 4.4	<0.001	27.4 ± 4.9	25.0 ± 4.7	24.0 ± 4.4	<0.001	27.8 ± 4.2	27.2 ± 3.9	27.3 ± 3.8	0.236
WC, cm	94 ± 14	90 ± 14	89 ± 16	<0.001	89 ± 14	82 ± 13	79 ± 13	<0.001	99 ± 12	97 ± 12	98 ± 14	0.218
WHtR	0.57 ± 0.08	0.53 ± 0.08	0.52 ± 0.09	<0.001	0.56 (0.11)	0.49 (0.11)	0.47 (0.09)	<0.001	0.57 (0.09)	0.55 (0.08)	0.56 (0.09)	<0.001
WHR	0.89 (0.14)	0.85 (0.14)	0.86 (0.16)	<0.001	0.83 (0.09)	0.79 (0.08)	0.78 (0.09)	<0.001	0.95 (0.08)	0.92 (0.09)	0.93 (0.09)	<0.001
Energy intake adjusted to ΒMR	1.62 (0.38)	1.42 (0.30)	1.41 (0.24)	<0.001	1.66 (0.31)	1.44 (0.31)	1.44 (0.24)	<0.001	1.58 (0.44)	1.41 (0.29)	1.40 (0.24)	<0.001
EAT-LDP, 0–42 units	16.5 (13.0)	18.8 (6.3)	20.4 (6.3)	<0.001	8.6 (11.5)	19.6 (6.3)	22.7 (6.3)	<0.001	16.5 (14.6)	18.8 (6.3)	18.8 (6.3)	<0.001
Adherence to EAT-LDP, % low	72.5	45.0	39.8	<0.001	76.4	45.2	35.7	<0.001	68.0	44.7	43.6	<0.001
MedDietScore, 0–55 units	25.7 (3.0)	26.8 (2.8)	26.8 (3.0)	<0.001	26.3 (3.3)	28.0 (2.4)	28.3 (2.1)	<0.001	25.1 (2.7)	25.7 (1.9)	25.7 (1.9)	<0.001
Adherence to MDP, % low	80.3	54.9	54.1	<0.001	68.9	29.5	21.2	<0.001	93.4	81.3	84.5	<0.001
Smoking, % ever	52.1	60.1	51.8	0.001	34.7	51.3	42.3	<0.001	72.1	69.2	60.6	0.007
Physically inactivity, %	61.4	60.6	56.5	0.157	67.1	61.5	57.4	0.066	54.8	59.6	55.6	0.401

Continuous variables are presented as means ± standard deviations or medians (interquartile ranges), based on the normality of the variables’ distributions. Categorical variables are expressed as relative frequencies (%). Between-group comparisons were assessed using ANOVA or Kruskal–Wallis tests for continuous variables and the chi-square test for categorical data. Low income classification was assigned to individuals whose three-year mean annual income was <€10,000. The socio-economic status was assessed as an aggregate estimate of educational attainment and mean annual income over the past three years; this estimate was further categorized as low class (income ≤ €8000 and educational level < 14 years; income ≤ €8000 or €8001–10,000 and educational level < 9 years), high class (income > €20,000 and educational level 10–14 years; income €10,001–20,000 or >€20,000 and educational level ≥ 15 years), and middle class (all other cases). Residential areas were classified as urban or suburban according to the Hellenic Statistical Authority, with urban municipalities or communes defined as those where the largest settlement has >2000 inhabitants. The classification of body weight status followed WHO-recommended BMI thresholds: <18.5 kg/m^2^ (underweight), 18.5–24.9 kg/m^2^ (normal), 25.0–29.9 kg/m^2^ (overweight), and ≥30.0 kg/m^2^ (obesity) [27]. Sex-based central obesity thresholds were determined as WC ≥88 cm and ≥102 cm for females and males, respectively [28]. An abnormal WHtR was defined as ≥0.50 for both sexes [29], while elevated WHR was defined as ≥0.80 and ≥0.95 for females and males, respectively [30]. Adherence to the EAT-LDP and MDP was considered low when the corresponding dietary scores fell below the sample-specific medians, i.e., EAT-LDP < 17/42 and MedDietScore < 26/55, respectively. Abbreviations: BMI: body mass index, BMR: basal metabolic rate, hs-CRP: high-sensitivity C-reactive protein, EAT-LDP: EAT-Lancet diet pattern, MDP: Mediterranean dietary pattern, WC: waist circumference, WHR: waist-to-hip ratio, WHtR: waist-to-height ratio.

**Table 3:** Results from nested Cox proportional hazards models assessing the relationship between educational level and 20-year CVD incidence among female and male participants of the ATTICA Study (*n* = 1988).

	Model 1	Model 2	Model 3	Model 4
Females				
Educational level, per 1 year	0.81 (0.77, 0.84) ***	0.99 (0.94, 1.06)	1.02 (0.95, 1.08)	1.02 (0.95, 1.09)
Age, per 1 year		1.30 (1.25, 1.35) ***	1.28 (1.23, 1.34) ***	1.21 (1.16, 1.27) ***
Hypertension, ref: normal			1.35 (0.78, 2.33)	1.37 (0.78, 2.42)
Hypercholesterolemia, ref: normal			3.72 (2.30, 6.02) ***	3.28 (2.00, 5.39) ***
Diabetes mellitus, ref: normal			8.41 (1.78, 39.8) **	9.89 (2.05, 47.7) **
WHtR, per 1 unit			1.30 (0.07, 25.1)	0.79 (0.04, 16.7)
Smoking, ref: never smokers				0.77 (0.46, 1.27)
EAT-LDP, per 1/42 unit				0.85 (0.79, 0.92) ***
Physically inactivity, ref: yes				1.13 (0.68, 1.90)
*p*-value (omnibus test)	<0.001	<0.001	<0.001	<0.001
*p*-value (likelihood ratio test)	<0.001	<0.001	<0.001	<0.001
Males				
Educational level, per 1 year	0.89 (0.86, 0.93) ***	0.97 (0.92, 1.03)	0.99 (0.93, 1.05)	0.99 (0.93, 1.05)
Age, per 1 year		1.29 (1.24, 1.34) ***	1.27 (1.23, 1.33) ***	1.21 (1.16, 1.27) ***
Hypertension, ref: normal			1.35 (0.87, 2.09)	1.43 (0.91, 2.24)
Hypercholesterolemia, ref: normal			1.69 (1.11, 2.57) *	1.90 (1.23, 2.93) **
Diabetes mellitus, ref: normal			4.27 (1.70, 10.7) **	4.78 (1.84, 12.4) **
WHtR, per 1 unit			2.21 (0.10, 47.6)	2.41 (0.09, 67.1)
Smoking, ref: never smokers				1.42 (0.92, 2.19)
EAT-LDP, per 1/42 unit				0.84 (0.78, 0.90) ***
Physically inactivity, ref: yes				0.97 (0.92, 2.19)
*p*-value (omnibus test)	<0.001	<0.001	<0.001	<0.001
*p*-value (likelihood ratio test)	<0.001	<0.001	0.002	<0.001

Results are presented as HR (95% CI). To evaluate improvements in the model fit relative to a null model, the statistical significance of the omnibus test was determined. Furthermore, the likelihood ratio test’s statistical significance suggested that the model fit improved as a result of the addition of each variable block in the model. Model 1: Educational level. Model 2: Model 1 + Age. Model 3: Model 2 + Hypertension + Hypercholesterolemia + Diabetes mellitus + WHtR. Model 4: Model 3 + Smoking + Adherence to EAT-LDP + Physical inactivity. *** *p*-value < 0.001, ** *p*-value < 0.01, * *p*-value < 0.05. Abbreviations: EAT-LDP: EAT-Lancet diet pattern, HR: hazards ratio, WHtR: waist-to-height ratio, 95% CI: 95% confidence interval.

**Table 4:** Results from the GSEM investigating the multifactorial interplay between education and the 20-year CVD incidence among participants of the ATTICA Study (*n* = 1988).

Outcome Variables	Manifest Variables	Path OR (95% CI)
20-year CVD incidence, ref.: no		
	Age at baseline, per 1 year	0.29 (0.24, 0.33) ***
	Hypertension, ref.: no	0.83 (0.38, 1.29) ***
	Hypercholesterolemia, ref.: no	1.06 (0.56, 1.56) ***
	Diabetes mellitus, ref.: no	1.43 (0.84, 2.01) ***
	BMI, per 1 kg/m^2^	−0.02 (−0.07, 0.03)
	Educational level, per 1 year	−0.02 (−0.08, 0.05)
Hypertension, ref.: no		
	Age at baseline, per 1 year	0.012 (0.010, 0.015) ***
	Smoking, ref.: never smokers	0.02 (−0.02, 0.07)
	EAT-LDP, per 1/42 unit	−0.007 (−0.012, −0.002) *
	Physically inactivity, ref.: yes	−0.04 (−0.085, −0.001) *
Hypercholesterolemia, ref.: no		
	Age at baseline, per 1 year	0.005 (0.003, 0.007) ***
	Smoking, ref: never smokers	0.048 (0.006, 0.090) *
	EAT-LDP, per 1/42 unit	−0.012 (−0.017, −0.007) ***
	Physically inactivity, ref.: yes	−0.03 (−0.07, 0.02)
Diabetes mellitus, ref.: no		
	Age at baseline, per 1 year	0.007 (0.005, 0.009) ***
	Smoking, ref.: never smokers	−0.009 (−0.050, 0.032)
	EAT-LDP, per 1/42 unit	−0.012 (−0.016, −0.007) ***
	Physically inactivity, ref.: yes	−0.06 (−0.10, −0.01) **
BMI, per 1 kg/m^2^		
	Age at baseline, per 1 year	0.005 (−0.019, 0.030)
	Smoking, ref.: never smokers	0.40 (−0.02, 0.82)
	EAT-LDP, per 1/42 unit	−0.027 (−0.079, 0.026)
	Physically inactivity, ref.: yes	0.003 (−0.441, 0.447)
EAT-LDP, per 1/42 unit		
	Educational level, per 1 year	0.45 (0.40, 0.50) ***
Smoking, ref.: never smokers		
	Educational level, per 1 year	0.001 (−0.004, 0.006)
Physically inactivity, ref.: yes		
	Educational level, per 1 year	0.009 (0.004, 0.013) ***

*** *p*-value < 0.001, ** *p*-value < 0.01, * *p*-value < 0.05. Abbreviations: BMI: body mass index, EAT-LDP: EAT-Lancet diet pattern, OR: odds ratios, 95% CI: 95% confidence interval.

## Data Availability

Data described in the manuscript, code book, and analytic code will be made available upon request to the corresponding author. Data are not publicly available due to being a part of an ongoing study.

## Abbreviations

The following abbreviations are used in this manuscript:

CVD	Cardiovascular diseases
BMI	Body mass index
BMR	Basal metabolic rate
DALYs	Disability-adjusted life years
EAT-LDP	EAT-Lancet diet pattern
FFQ	Food frequency questionnaire
GSEM	Generalized structural equation model
HR	Hazards ratio
hs-CRP	High-sensitivity C-reactive protein
ICD	International Classification of Diseases
OR	Odds ratio
RR	Risk ratio
RRR	Ratio of relative risks
SD	Standard deviation
SDI	Socio-demographic index
WC	Waist circumference
WHO	World Health Organization
WHtR	Waist-to-height ratio
WHR	Waist-to-hip ratio
95% CI	95% confidence interval

## References

[1] GBD 2021 Diseases Injuries Collaborators (2024). Global incidence, prevalence, years lived with disability (YLDs), disability-adjusted life-years (DALYs), and healthy life expectancy (HALE) for 371 diseases and injuries in 204 countries and territories and 811 subnational locations, 1990–2021: A systematic analysis for the Global Burden of Disease Study 2021. Lancet.

[2] Powell-Wiley T.M., Baumer Y., Baah F.O., Baez A.S., Farmer N., Mahlobo C.T., Pita M.A., Potharaju K.A., Tamura K., Wallen G.R. (2022). Social Determinants of Cardiovascular Disease. Circ. Res..

[3] Kubota Y., Heiss G., MacLehose R.F., Roetker N.S., Folsom A.R. (2017). Association of Educational Attainment with Lifetime Risk of Cardiovascular Disease: The Atherosclerosis Risk in Communities Study. JAMA Intern. Med..

[4] Jeong C., Lee K.N., Jung J.H., Sohn T.S., Kwon H.S., Han K., Lee S.H. (2025). Socioeconomic gradients and inequalities in all-cause mortality and cardiovascular diseases: A retrospective cohort study using Korean NHANES-mortality linkage data. Public Health.

[5] Lee J.R., Paultre F., Mosca L. (2005). The association between educational level and risk of cardiovascular disease fatality among women with cardiovascular disease. Women’s Health Issues Off. Publ. Jacobs Inst. Women’s Health.

[6] Khaing W., Vallibhakara S.A., Attia J., McEvoy M., Thakkinstian A. (2017). Effects of education and income on cardiovascular outcomes: A systematic review and meta-analysis. Eur. J. Prev. Cardiol..

[7] Backholer K., Peters S.A.E., Bots S.H., Peeters A., Huxley R.R., Woodward M. (2017). Sex differences in the relationship between socioeconomic status and cardiovascular disease: A systematic review and meta-analysis. J. Epidemiol. Community Health.

[8] Magnani J.W., Ning H., Wilkins J.T., Lloyd-Jones D.M., Allen N.B. (2024). Educational Attainment and Lifetime Risk of Cardiovascular Disease. JAMA Cardiol..

[9] Merz E.C., Myers B., Hansen M., Simon K.R., Strack J., Noble K.G. (2024). Socioeconomic Disparities in Hypothalamic-Pituitary-Adrenal Axis Regulation and Prefrontal Cortical Structure. Biol. Psychiatry Glob. Open Sci..

[10] de Mestral C., Stringhini S. (2017). Socioeconomic Status and Cardiovascular Disease: An Update. Curr. Cardiol. Rep..

[11] WHO, FAO (2019). Sustainable Healthy Diets-Guiding Principles.

[12] Willett W., Rockstrom J., Loken B., Springmann M., Lang T., Vermeulen S., Garnett T., Tilman D., DeClerck F., Wood A. (2019). Food in the Anthropocene: The EAT-Lancet Commission on healthy diets from sustainable food systems. Lancet.

[13] IPCC, Intergovernmental Panel on Climate Change (2019). Food security. Climate Change and Land: IPCC Special Report on Climate Change, Desertification, Land Degradation, Sustainable Land Management, Food Security, and Greenhouse Gas Fluxes in Terrestrial Ecosystems.

[14] World Health Organization Gender. https://www.who.int/health-topics/gender#tab=tab_1.

[15] Damigou E., Kouvari M., Chrysohoou C., Barkas F., Kravvariti E., Pitsavos C., Skoumas J., Michelis E., Liberopoulos E., Tsioufis C. (2023). Lifestyle Trajectories Are Associated with Incidence of Cardiovascular Disease: Highlights from the ATTICA Epidemiological Cohort Study (2002–2022). Life.

[16] Panagiotakos D., Georgousopoulou E., Notara V., Pitaraki E., Kokkou E., Chrysohoou C., Skoumas Y., Metaxa V., Pitsavos C., Stefanadis C. (2016). Education status determines 10-year (2002–2012) survival from cardiovascular disease in Athens metropolitan area: The ATTICA study, Greece. Health Soc. Care Community.

[17] Pitsavos C., Panagiotakos D.B., Chrysohoou C., Stefanadis C. (2003). Epidemiology of cardiovascular risk factors in Greece: Aims, design and baseline characteristics of the ATTICA study. BMC Public Health.

[18] Panagiotakos D.B., Pitsavos C., Chrysohoou C., Skoumas I., Stefanadis C. (2008). Five-year incidence of cardiovascular disease and its predictors in Greece: The ATTICA study. Vasc. Med..

[19] Katsouyanni K., Rimm E.B., Gnardellis C., Trichopoulos D., Polychronopoulos E., Trichopoulou A. (1997). Reproducibility and relative validity of an extensive semi-quantitative food frequency questionnaire using dietary records and biochemical markers among Greek schoolteachers. Int. J. Epidemiol..

[20] Stubbendorff A., Sonestedt E., Ramne S., Drake I., Hallstrom E., Ericson U. (2022). Development of an EAT-Lancet index and its relation to mortality in a Swedish population. Am. J. Clin. Nutr..

[21] Panagiotakos D.B., Pitsavos C., Stefanadis C. (2006). Dietary patterns: A Mediterranean diet score and its relation to clinical and biological markers of cardiovascular disease risk. Nutr. Metab. Cardiovasc. Dis..

[22] Papathanasiou G., Georgoudis G., Papandreou M., Spyropoulos P., Georgakopoulos D., Kalfakakou V., Evangelou A. (2009). Reliability measures of the short International Physical Activity Questionnaire (IPAQ) in Greek young adults. Hellenic. J. Cardiol..

[23] Georgoulis M., Damigou E., Chrysohoou C., Barkas F., Kravvariti E., Tsioufis C., Pitsavos C., Liberopoulos E., Sfikakis P.P., Panagiotakos D.B. (2024). Increased body weight and central adiposity markers are positively associated with the 20-year incidence of cardiovascular disease: The ATTICA epidemiological study (2002–2022). Nutr. Res..

[24] Chrysohoou C., Panagiotakos D.B., Pitsavos C., Das U.N., Stefanadis C. (2004). Adherence to the Mediterranean diet attenuates inflammation and coagulation process in healthy adults: The ATTICA Study. J. Am. Coll. Cardiol..

[25] Lloyd-Jones D.M., Huffman M.D., Karmali K.N., Sanghavi D.M., Wright J.S., Pelser C., Gulati M., Masoudi F.A., Goff D.C. (2017). Estimating longitudinal risks and benefits from cardiovascular preventive therapies among Medicare patients: The million hearts longitudinal ASCVD risk assessment tool: A special report from the American heart association and American College of cardiology. J. Circ..

[26] Beiser A., D’Agostino R.B., Seshadri S., Sullivan L.M., Wolf P.A. (2000). Computing estimates of incidence, including lifetime risk: Alzheimer’s disease in the Framingham Study. The Practical Incidence Estimators (PIE) macro. Stat. Med..

[27] Kushner R.F. (2012). Clinical assessment and management of adult obesity. Circulation.

[28] Alberti K.G., Eckel R.H., Grundy S.M., Zimmet P.Z., Cleeman J.I., Donato K.A., Fruchart J.C., James W.P., Loria C.M., Smith S.C. (2009). Harmonizing the metabolic syndrome: A joint interim statement of the International Diabetes Federation Task Force on Epidemiology and Prevention; National Heart, Lung, and Blood Institute; American Heart Association; World Heart Federation; International Atherosclerosis Society; and International Association for the Study of Obesity. Circulation.

[29] Browning L.M., Hsieh S.D., Ashwell M. (2010). A systematic review of waist-to-height ratio as a screening tool for the prediction of cardiovascular disease and diabetes: 0·5 could be a suitable global boundary value. Nutr. Res. Rev..

[30] WHO (2011). Waist Circumference and Waist-Hip Ratio: Report of a WHO Expert Consultation.

[31] Sigala E.G., Vaina S., Chrysohoou C., Dri E., Damigou E., Tatakis F.P., Sakalidis A., Barkas F., Liberopoulos E., Sfikakis P.P. (2024). Sex-related differences in the 20-year incidence of CVD and its risk factors: The ATTICA study (2002–2022). Am. J. Prev. Cardiol..

[32] Alemu Y.M., Bagheri N., Wangdi K., Richardson A., Chateau D.J.C.P.H. (2025). Disparities in primary cardiovascular risks and social determinants: Multilevel analysis of national surveys. Crit. Public Health.

[33] Petrelli A., Sebastiani G., Di Napoli A., Macciotta A., Di Filippo P., Strippoli E., Mirisola C., d’Errico A. (2022). Education inequalities in cardiovascular and coronary heart disease in Italy and the role of behavioral and biological risk factors. Nutr. Metab. Cardiovasc. Dis..

[34] Powell K.L., Stephens S.R., Stephens A.S. (2021). Cardiovascular risk factor mediation of the effects of education and Genetic Risk Score on cardiovascular disease: A prospective observational cohort study of the Framingham Heart Study. BMJ Open.

[35] Kontele I., Panagiotakos D., Yannakoulia M., Vassilakou T. (2025). Socio-Demographic Determinants of Mediterranean Diet Adherence: Results of the EU-National Health Interview Survey (EHIS-3). J. Hum. Nutr. Diet. Off. J. Br. Diet. Assoc..

[36] Ferreira A.F., Abreu S., Liz Martins M. (2024). Determinants of adherence to sustainable healthy diets among Portuguese adults. NFS J..

[37] Carvalho C., Correia D., Lopes C., Torres D. (2025). Adherence to the EAT-Lancet Planetary Health Diet in Portugal and its associations with socioeconomic and lifestyle factors. Eur. J. Nutr..

[38] Boujelbane M.A., Ammar A., Salem A., Kerkeni M., Trabelsi K., Bouaziz B., Masmoudi L., Heydenreich J., Schallhorn C., Müller G. (2025). Sex-Specific Insights into Adherence to Mediterranean Diet and Lifestyle: Analysis of 4000 Responses from the MEDIET4ALL Project. Front. Nutr..

[39] Liu J., Shen Q., Wang X. (2024). Emerging EAT-Lancet planetary health diet is associated with major cardiovascular diseases and all-cause mortality: A global systematic review and meta-analysis. Clin. Nutr..

[40] Sawicki C.M., Ramesh G., Bui L., Nair N.K., Hu F.B., Rimm E.B., Stampfer M.J., Willett W.C., Bhupathiraju S.N. (2024). Planetary health diet and cardiovascular disease: Results from three large prospective cohort studies in the USA. Lancet Planet Health.

[41] Zhang S., Dukuzimana J., Stubbendorff A., Ericson U., Borne Y., Sonestedt E. (2023). Adherence to the EAT-Lancet diet and risk of coronary events in the Malmo Diet and Cancer cohort study. Am. J. Clin. Nutr..

[42] Zhang S., Marken I., Stubbendorff A., Ericson U., Qi L., Sonestedt E., Borne Y. (2024). The EAT-Lancet Diet Index, Plasma Proteins, and Risk of Heart Failure in a Population-Based Cohort. JACC Heart Fail.

[43] Zhang S., Stubbendorff A., Ericson U., Wandell P., Niu K., Qi L., Borne Y., Sonestedt E. (2023). The EAT-Lancet diet, genetic susceptibility and risk of atrial fibrillation in a population-based cohort. BMC Med..

[44] Ibsen D.B., Christiansen A.H., Olsen A., Tjonneland A., Overvad K., Wolk A., Mortensen J.K., Dahm C.C. (2022). Adherence to the EAT-Lancet Diet and Risk of Stroke and Stroke Subtypes: A Cohort Study. Stroke.

[45] Shan Y., Bertrand K.A., Petrick J.L., Sheehy S., Palmer J.R. (2025). Planetary Health Diet Index in relation to mortality in a prospective cohort study of United States Black females. Am. J. Clin. Nutr..

[46] Martins L.B., Gamba M., Stubbendorff A., Gasser N., Lobl L., Stern F., Ericson U., Marques-Vidal P., Vuilleumier S., Chatelan A. (2025). Association between the EAT-Lancet Diet, Incidence of Cardiovascular Events, and All-Cause Mortality: Results from a Swiss Cohort. J. Nutr..

[47] Guzman-Castellanos K.B., Zazpe I., Santiago S., Bes-Rastrollo M., Martinez-Gonzalez M.A. (2024). Planetary Health Diet and Cardiovascular Disease Risk in the Seguimiento Universidad de Navarra (SUN) Cohort. Nutrients.

[48] Colizzi C., Harbers M.C., Vellinga R.E., Verschuren W.M.M., Boer J.M.A., Biesbroek S., Temme E.H.M., van der Schouw Y.T. (2023). Adherence to the EAT-Lancet Healthy Reference Diet in Relation to Risk of Cardiovascular Events and Environmental Impact: Results From the EPIC-NL Cohort. J. Am. Heart Assoc..

